# Triple negative breast cancer: Pitfalls and progress

**DOI:** 10.1038/s41523-022-00468-0

**Published:** 2022-08-20

**Authors:** Paola Zagami, Lisa Anne Carey

**Affiliations:** 1 Department of Oncology and Hematology, University of Milano, Milan, Italy; 2 Lineberger Comprehensive Cancer Center, University of North Carolina at Chapel Hill, Chapel Hill, NC USA

**Keywords:** Breast cancer, Breast cancer

## Abstract

Triple negative breast cancer (TNBC) is characterized by the lack of estrogen and progesterone receptor expression and lacks HER2 overexpression or gene amplification. It accounts for 10–15% of incident breast cancers and carries the worst prognosis. TNBC is overrepresented among Black and pre-menopausal women and is associated with significant psychological and treatment-related burdens, including financial toxicity. Like other breast cancers, TNBC is biologically heterogeneous, leading to diverse clinical and epidemiological behaviors, however, unlike the other clinical subtypes, in TNBC we still lack tumor-specific targeted therapy. Early TNBC outcomes have improved due to the intensification of therapies, including improvements in polychemotherapy and the addition of immunotherapy. Future efforts are needed to identify targetable aberrations for specific drug therapy, prevent immune evasion, and increase social-economic support. Given that the name TNBC illustrates its lack of specifically targeted and effective therapy, we look forward to being able to retire the name in favor of a group of targetable entities within what is now called “TNBC”.

## Introduction

Triple negative breast cancer (TNBC), which accounts for 15–20% of incident breast cancers, is the only breast cancer (BC) subtype that lacks targeted treatments. Using clinical assays, TNBC is human epidermal growth factor receptor 2 (HER2) negative and has <1% expression of estrogen receptors (ER) and progesterone receptors (PR) by immunostaining. It is a biologically aggressive tumor, characterized by moderate/high grade and highly proliferative cancer cells, which, together with limited treatment options leads to the poorest prognosis among BC subtypes. TNBC presents most commonly as an invasive ductal carcinoma; however, there are special TNBC histologies that warrant special attention due to different biology and prognosis^1^. For example, low grade adenoid cystic and secretory TNBC carry better prognosis and, unless high risk clinical features exist, may not require systemic chemotherapy^2^. Chemotherapy remains the chief systemic treatment for TNBC despite multiple efforts to discover targetable therapeutic abnormalities. The lack of progress in finding tailored agents for TNBC stands in stark contrast to multiple agents targeting ER and HER2 in those clinical subtypes. With that said, there are notable recent novel therapeutic advances. These include poly adenosine diphosphate–(ADP)-ribose polymerase inhibitors (PARPi) for patients with germline BRCA1/2 mutations (which is especially relevant for TNBC), FDA-approved in 2019 for metastatic disease then in 2021 for early disease^3–5^ as well as the first immunotherapy regimens for TNBC, also approved for metastatic disease beginning in 2019 and for early disease in 2021^6,7^.

This review is based on a plenary presentation given at the AACR-San Antonio Breast Cancer Research Symposium (AACR-SABCS) in December 2021, in which we describe the historical overview of TNBC, the current therapeutic landscape, and efforts in the development of new treatments that we anticipate will result in improved prognosis and patient, community, and population health impacts.

## History of TNBC

Twenty years ago, new molecular taxonomy using gene expression profiling illustrated that the BC represents several distinct biologic entities that are only in part recapitulated by ER- and HER2 clinical assays. Existing intrinsic and other molecular subtypes of BC have been identified to carry different prognoses and possibly treatment responsiveness; future profiling efforts doubtless with further illuminate this heterogeneity.

### Intrinsic molecular subtypes in TNBC

**Fig. 1:**
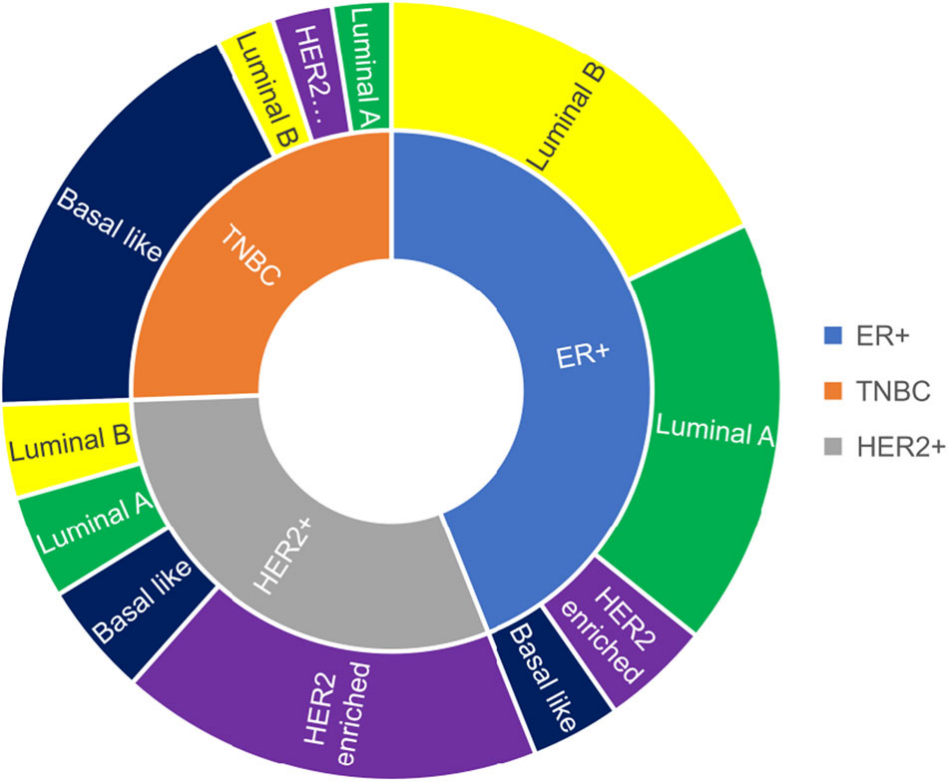
Intrinsic molecular subtypes of breast cancer. Within each clinical subtype there are multiple molecular subtypes. ER endocrine receptor; TNBC triple negative breast cancer; HER2 Human Epidermal Growth Factor Receptor 2.

Distinct molecular portraits of breast cancer, originally identified as Luminal, HER2-Enriched, Basal-like (BL), and Normal-like breast cancer, were based unsupervised gene expression analysis. Luminal A and B subtypes express keratins 8/18 and ER-related gene clusters, the BL has overexpression of keratin 5, 17 and epithelial grown factor receptors (EGFR)-related genes, and the HER2-Enriched subtype is characterized by expression of Erb-B2-related genes^8^; each of these molecular subtypes can be found within clinical subsets (Fig. 1). About 80% of TNBC are BL, and BL tumors cluster biologically far from the other BC subtypes, making intrinsic subtyping less useful for meaningful subclassification than in the other clinical subtypes^9^. More detailed confirmation of BC heterogeneity has come from multiple efforts to examine DNA, RNA, microRNA, and protein expression patterns through cross-platform analyses such as The Cancer Genome Atlas and METABRIC^10,11^.

Within-TNBC transcriptomic efforts have further elucidated several molecularly-defined entities. Lehmann and colleagues initially described seven subtypes of TNBC, based on specific clusters of gene expression potentially useful for targeted therapies. These included basal-like (BL1 and BL2) subtypes enriched for proliferation genes, the immunomodulatory (IM) subtype overexpressing immune signaling genes, luminal androgen receptor (LAR) with AR-activated gene expression, mesenchymal (M) and mesenchymal stem-like (MSL) subtypes characterized by cell motility and angiogenesis-related gene expression, respectively, and an unstable subtype not further characterizable^12^. These TNBC subclassifications were derived from analyzing bulk surgical samples including both cancer and non-cancer cells creating the tumor environment. Deeper understanding of the immunomodulatory and the MSL subtypes, which reflected not only intrinsic characteristics of cancer cells but also extrinsic signals/elements, including immune and stromal cells, led those investigators to exclude them, thus refining the Lehman cancer classifications into four TNBC types (BL1,2, LAR and M)^13^. In a similar within-TNBC effort on 198 tumors, the Brown lab found four subtypes (LAR, MSL, BL-immunosuppressed, and BL-immunoactivated), also with potential subtype-specific therapeutic implications^14^.

### Clinical implications of subtypes in TNBC

The introduction into clinical practice of RNA-based assays that subtype BC by intrinsic biology and prognosis is well established for clinical decision-making in early ER+BC, and recent studies suggest potential value in metastatic, and in clinically HER2-positive disease^15–19^.

In TNBC, intrinsic subtype profiling at this point has less clear clinical implications. Shepherd and colleagues analyzed pre-treatment early TNBC from CALGB 40603, a neoadjuvant clinical trial testing bevacizumab and/or carboplatin added to a standard anthracycline/paclitaxel regimen, evaluating long term outcomes and possible genomic predictors of outcomes. The investigators did not find significant value of molecular profiling in identifying the benefit of adding platinum agents or in outcome. Although patients whose tumors appeared BL-immuneactivated achieved a higher pathological complete response (pCR) rate, this improvement did not translate into improved event-free survival (EFS)^20^. Similar results were also seen in the metastatic setting. In the TNT trial testing carboplatin versus docetaxel in TNBC in the first-line setting, most TNBC were BL and while non-BL appeared to benefit more from the taxane, these occurred infrequently so are unlikely to be clinically meaningful. Only those with gBRCA1/2 were particularly sensitive to the platinum agent, with 6.8 months of median PFS (vs 4.4m with docetaxel)^21^.

The correlation between TNBC and the BL subtype was further defined in a large cohort of 412 TN and 473 BL samples of BC that were molecularly characterized, confirming that triple negative was largely but not exclusively basal-like (60–80%) and that 70% of BL subtype were TNBC. DNA aberrations typical of TNBC were also examined, including *TP53* mutations found in more than 80%, *MYK* amplification in about half, *Rb1* loss in 20%, *PIK3CA* amplifications in 30%, and BRCA1/2 functional loss in about 20%. Among all these aberrations, only *PIK3CA* and *BRCA1/2* can be considered clinically actionable ESCAT I or II abnormalities^22^. However, valuable biologic information is not limited to DNA and RNA, new data are emerging from proteomic/proteogenomic studies that may aid in identifying therapeutically-relevant vulnerabilities in TNBC^23,24^.

## Tumor Microenvironment of TNBC

The initial and subsequent modification of the Lehman classifier performed on bulk tumor described above highlighted that non-cancer cells contribute significantly to gene expression profiles and modify treatment response and prognosis of TNBC. The dynamic interactions between cancer cells and the immune system, originally referred to as “immune surveillance”, illustrate the importance that each component of the tumor microenvironment has on behavior and prognosis. Over time, this theory has been extended to the tumor “immunoediting” model which includes three phases: the elimination phase, in which the innate and adaptive immune system identifies and destroys tumor cells (the real “immune surveillance”); the equilibrium phase, in which the immune system maintains control over cancer cells with a balance between the production of immunostimulants (e.g., interleukin [IL] 12) and immunosuppressants (e.g., IL23) and the escape phase, in which the tumor cells escape suppression by the immune system, leading to tumor progression^25^. Since these findings, the ability to evade immune suppression, involving all these external cellular and humoral elements, has become a hallmark of cancer^26^. BC is less immune-activated than many other “hot” tumor types, such as melanoma, bladder or some lung cancers, in part related to fewer immunogenic tumor antigens and non-synonymous (amino acid-altering) mutations^27^. Even if TNBC is the most immune-activated subtype among all BC, as determined by not only immune gene expression but also levels of intra-tumoral and stromal tumor-infiltrating lymphocytes (TILs)^28^, not all patients benefit from immunotherapy. How best to define immune activation is a work in progress; current approaches include the presence of TILs, expression of the programmed death-ligand 1 (PDL1) protein, immune gene signatures, RNA expression of individual immune genes, and immune cell clonality (Tcells and Bcells) studies.

### Tumor infiltrating lymphocytes (TILs)

**Fig. 2:**
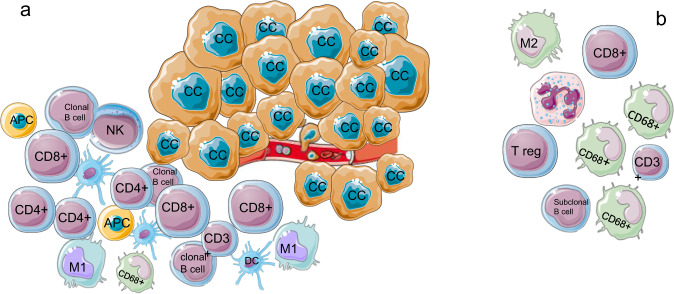
Tumor microenvironment. **a** Immune-stimulating microenvironment with homogeneous distribution of CD8+ and CD4+ T cells, M1 polarized macrophages, APC and dendritic cells, in proximity with cancer cells. **b** Immune-escaping microenvironment with higher ratio of CD68+:CD3+ T cells, and M2 polarized macrophages, and more distance between cancer and immune cells. CD4+and CD8+:Tcells marker; CD68+: pan-macrophage marker; APC: antigen presenting cells; CC: cancer cells; DC: dendritic cells. Figure includes modified templates from Servier Medical Art (smart.servier.com).

TILs include many types of immune cells infiltrating the tumor with varying density and localization. The most representative are CD8+ T cells, the main effectors of the immune response, CD4+ T helper cells, natural killer cells, M1 macrophages, and dendritic cells (DCs). Regulatory T cells, M2 macrophages, and immature DCs can conversely create an immune escape tumor microenvironment that correlates with worse prognosis^29^ (Fig. 2). Stromal and epithelial TILs, easily measured in hematoxylin and eosin (H&E)-stained slides, vary widely within TNBC and their activity in the immune-surveillance are influenced by individual immune cell gene expression, soluble factors such as interferon release, and somatic, epigenetic or germline mutations^30^.

Their presence in the tumor microenvironment has been validated as an independent good prognostic factor in both chemotherapy-pretreated and untreated patients with early TNBC^28,31–33^.Quantitative levels of TILs correlate linearly with lower risk for recurrence or death. In a large cohort of patients with TNBC treated with adjuvant chemotherapy, each 10% of increased TILs (intra-tumoral or stromal) corresponded to an approximately 15% reduction of risk for recurrence^28^. Higher TILs also predicts response to neoadjuvant chemotherapy. In the subgroup of TNBC within a large pooled analysis of 3771 samples from neoadjuvant trials, tumors with high TILs (>60% immune cells in the stromal tissue within the tumor) achieved 50% pathologic complete response (pCR) rate compared with 31% in those with lower TILs^32^. Moreover, higher pCR rates were observed in patients with high TILs in a trial adding carboplatin to a neoadjuvant anthracycline and taxane regimen versus the non-carboplatin, suggesting that TILs might predict benefit to specific chemotherapy agents, although this interaction was not significant in the TNBC subset^33^.

### Other immune biomarkers

In addition to known predictive biomarkers of clinical benefit from immunotherapy, such as PDL1 expression and DNA mismatch repair (MMR) alterations, newer biomarkers such as high tumor mutational burden (TMB)^34,35^ may prove useful in TNBC. Frequent genomic alterations seen in TNBC may contribute to altered immune response, such as *TP53* mutation or deletion of 17p that reduce innate immune signaling and immune T cell infiltration, respectively^36,37^. A subanalysis of TCGA and METABRIC data found that immune-enriched TNBC were more likely to have lower somatic copy number alteration, lower neoantigen burden, lower clonal heterogeneity, and better prognosis suggestive of effective immune surveillance and elimination^38^. The anti-cancer adaptive immune response is not mediated only by T cells. The presence of clonally (and not subclonal) expanded B cell TILs, enriched for B cell-receptors (BCR) somatic hypermutation, mostly observed in tumor microenvironment of BL and HER2-enriched subtypes from mRNA sequencing, is also related to improved outcomes^39,40^.

### Spatial approaches

Emerging data suggest that merely the number and the immune phenotype of TILs are not enough to define tumor immune activation, that spatial relationships among cancer and immune cells are equally important^41^. A recent study using laser capture microdissection gene expression profiles classified four different immune-tumor microenvironment (TME) subtypes^42^, including the “inflamed” subtype, characterized by CD8+ T cells distributed in both epithelial and stromal tumor compartments and high immune gene expression, compared with the less immune-activated margin and stroma restricted-TME subtypes, and the least immune-activated group, the “immune desert”, in which there were few CD8+ T cells at all, and only at the margin^42^.

Newer technologies that allow improved visualization of cellular compartments within a tumor, the importance of both cell type and localization in prognosis and prediction of drug benefit is becoming clearer^43^. One recent study of TNBC patients treated with neoadjuvant chemotherapy found that PDL1 expression and closer spatial relationships of tumor cells to T cells independently predicted pCR^43^. In the phase 3 NeoTRIPaPDL1 trial of neoadjuvant chemotherapy plus atezolizumab or placebo, both cell features such as PDL1 expression as well as a high degree of spatial interaction between epithelial and microenvironmental cells appeared predictive of atezolizumab benefit^44^. Better understanding of the nature of not only the tumor cells, surrounding normal cells, immune cells and microenvironment, but their interactions may augment our ability to identify those patients who will benefit from the addition of immunotherapy to chemotherapy and exclude those patients who could have good outcomes with only chemotherapy and avoid toxicities seen with ICI.

## Clinical and relapse features

BC is the most common tumor and the main cause of cancer-related mortality worldwide; TNBC is about 15–20% among all. Epidemiology, distribution and mortality from TNBC varies within populations based on race or country of origin. In the prospective population-based study in North Carolina, the Carolina Breast Cancer Study (CBCS), designed to oversample Black and premenopausal women with newly diagnosed BC, it was observed that patients with TNBC were far more likely to be Black, younger than those with other subtypes, had tumors diagnosed at higher stage, and those tumors were mostly high-grade^45^. This racial and age distribution was confirmed and extended in CBCS3, in which intrinsic subtyping revealed that a young Black woman was more than twice (37 vs 15%) as likely to have a BL tumor (the intrinsic molecular subtype comprising the majority of TNBC) as an older white woman^46^.

The aggressive biological and clinical behavior of TNBC translates into more frequent and earlier relapses than other subtypes of BC. It is well established that the risk of early distant recurrence within five years of diagnosis is nearly three-fold higher for TNBC compared with non-TNBC^47^. Conversely, the risk of late relapse after 5 years is less than 3%^48^. The most common sites of relapses are lung, lymph nodes and brain (in which early or late involvement occurs in approximately 10 and 40%, respectively)^49^. Optimizing treatment for patients with brain metastases remains an unmet need.

## Recent advances in early TNBC

**Fig. 3:**
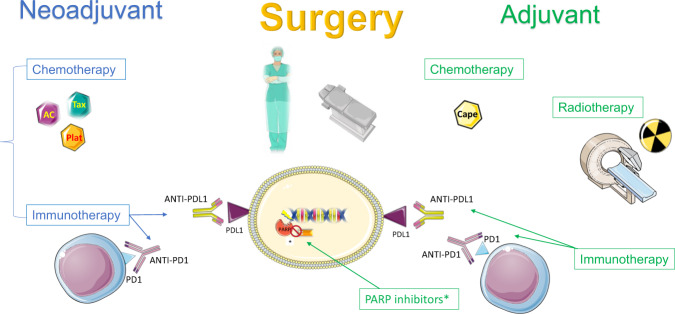
Current multidisciplinary treatment of TNBC, including neoadjuvant chemotherapy plus immunotherapy, surgery and radiation, and adjuvant treatment with ongoing immunotherapy. Adjuvant PARP inhibitors are given for those with germline BRCA1 or 2 mutations and capecitabine for those with residual disease, although optimal integration in the immunotherapy era is uncertain. In blue neoadjuvant treatment; in green adjuvant treatments. *only for germline carriers of BRCA1-2 mutations. Figure includes modified templates from Servier Medical Art (smart.servier.com).

The risk of relapse from TNBC has decreased with improved overall treatment and better chemotherapy regimens (Fig. 3), even in the pre-immunotherapy and capecitabine era. In a British Columbia registry, the risk of relapse from TNBC decreased by 25–40% between 1986–1992 and 2004–2008^43^. Chemotherapy advances, which improved outcomes among all BC patients, particularly improved outcomes in TNBC. This includes the sequential addition of taxanes^50^ and the introduction of dose-dense schedules^51^. The possibility to de-escalate to less toxic regimens omitting the anthracycline component in TNBC is still controversial. A benefit in 4-year invasive DFS of an anthracycline/taxane regimen (AC-T) versus taxane plus cyclophosphamide (TC, given for six cycles) was seen in the approximately 1300-patient TNBC subset of the phase III ABC trials. Counter to this, the small 100-patient NeoSTOP trial randomized patients to an anthracycline/taxane regimen versus taxane plus carboplatin, finding similar pCR rates, and a meta-analysis based on the PlanB/SUCCESS-C trials, which included a similar subset of TNBC patients as in the ABC trials, compared AC-T to six cycles of TC finding, no difference in five-year DFS^52–54^.

### Chemotherapy timing and tailoring

**Fig. 4:**
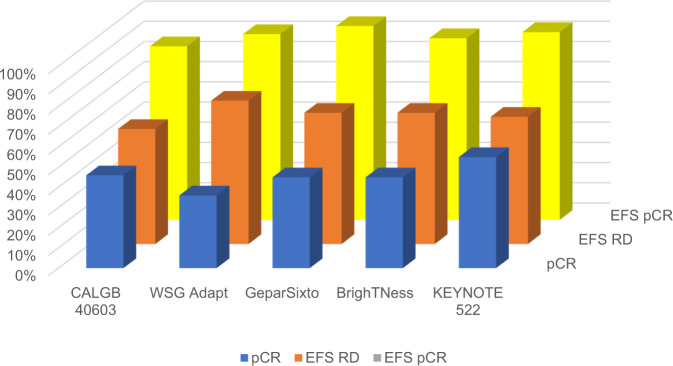
Relationship of pCR and RD with EFS in TNBC. pCR pathological complete response; RD residual disease; EFS event-free survival; TNBC triple negative breast cancer.

Among the most important recent advances for TNBC patients is the use of neoadjuvant chemotherapy, which increases the percentage of patients eligible for breast cancer conservation surgery and for de-escalation of axillary surgery^55^. In CALGB 40603, among TNBC patients with pre-treatment clinically positive nodes, 67% had pathologic nodal clearance, potentially avoiding axillary node dissection^56^. The benefit of neoadjuvant chemotherapy is not only in surgical outcomes but also in the ability to tailor therapy based on pCR. There is a particularly strong relationship in TNBC between pCR and increased EFS (Fig. 4).

As noted in Fig. 4, those patients with residual disease after chemotherapy-based neoadjuvant treatment still have an unacceptably high relapse rate, even in the more recent trials using ICI. The CREATE-X trial changed clinical practice for this group of patients by escalating post-neoadjuvant treatment by adding 4–6 cycles of adjuvant capecitabine, which resulted in an absolute benefit in the TNBC subset in terms of DFS and OS of 14 and 8%, respectively^57^. Similar results of adjuvant low dose capecitabine in TNBC were seen in SYSUCC-001 trial^58^. It should be noted that both studies enrolled Asian patients that have different dihydropyrimidine dehydrogenase genotype and phenotype than non-Asian population^59^. On the contrary, no improvement of DFS was observed adding adjuvant capecitabine after standard chemotherapy in a TNBC GEICAM trial^60^.

### PARP inhibitors

One example of targeted therapy relevant in early TNBC is the use of the PARP inhibitor olaparib in patients with germline mutations in *BRCA1/2* (gBRCA1/2) early BC. This class of drugs acts in tumors with loss of homologous recombination (HR), which is a *BRCA1/2*-dependent function. Through synthetic lethality, by inhibiting PARP-mediated single strand DNA repair, cells with HR-deficiency die. The OlympiA trial demonstrated a 9% difference in 3-year invasive disease-free survival (iDFS) in favor of 1 year of adjuvant olaparib vs placebo in patients with high risk HER2-negative and gBRCA1/2-positive BC who had completed standard chemotherapy. More than 80% of enrolled patients had TNBC^3^. Based on metastatic data, it is assumed but not yet proven that this effect would be seen also in the less-common germline PALB2 or somatic BRCA1/2 mutation-associated tumors^61^. PARP inhibitor (talazoparib) was studied also as neoadjuvant treatment in similar population of newly diagnosed TNBC, yielding 45% pCR rates with a single agent, similar to what would be expected with polychemotherapy (45%)^62^. In the TNBC subset of a small randomized phase II trial, GeparOla, paclitaxel plus olaparib had similar pCR rate, and better toxicity, as paclitaxel plus carboplatin in patients with early somatic or gBRCA1/2 mutated BC^63^. Additional efficacy was not seen by adding PARP inhibitors to standard chemotherapy, for example, veliparib combined with anthracycline/taxane/platinum chemotherapy in BrighTNess did not improve pCR or EFS^64^.

### Immune checkpoint inhibitors (ICI)

The most recent advance in early TNBC is the advantage in both pCR, and now demonstrated for EFS, of adding ICI to standard neoadjuvant chemotherapy. Unlike metastatic disease, the benefit of adding ICI is not limited to PDL1-positive tumors in early TNBC. KEYNOTE 522 was a large practice-changing trial that was unusual in that it was powered for both pCR and EFS endpoints. It enrolled patients with high risk early TNBC, most of whom were PDL1 positive, to receive four standard chemotherapy drugs with or without pembrolizumab and then postoperatively continue ICI in that arm^7^. The dual primary endpoints were met with an absolute benefit in terms of pCR and 3y-EFS of about 7%, at the cost of a higher percentage of grade 3 immune-related toxicities (15 vs 2%)^65^. The IMpassion 031 trial was consistent, demonstrating increased pCR by adding atezolizumab to nab-paclitaxel followed by anthracycline and cyclophosphamide (AC). In this trial, as in others, there was higher pCR rates in both chemotherapy alone and chemotherapy plus ICI arms in PDL1-positive than PDL1-negative. In other words, in TNBC, PDL1 is a predictive biomarker for both chemotherapy and ICI. The chemotherapy drug backbone and the timing, sequence, and duration of ICI administration may modify outcomes and treatment response; the optimal combination and endpoints are not yet clear. For example, in the small GeparNuevo the administration of durvalumab alone before start of chemotherapy appeared to provide the same benefit of ICI as in KEYNOTE 522, without the adjuvant ICI^66^. There are multiple ongoing trials also examining ICI plus chemotherapy in the adjuvant setting.

## Recent advances in metastatic TNBC

**Fig. 5:**
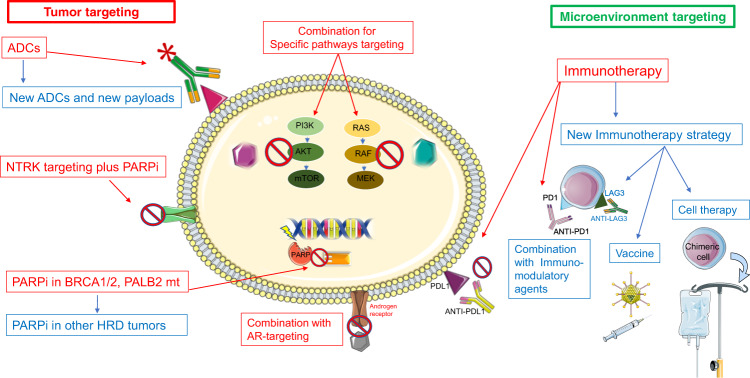
Therapeutic approaches in metastatic TNBC (red: current approaches, Blue: future directions). ADC antibody drug conjugate; PARPi: poly-ADP ribose polymerase inhibitors; HRD homologous recombination deficiency; AR androgen receptors. Figure includes modified templates from Servier Medical Art (smart.servier.com).

Unlike other subtypes and the early setting, few therapeutic approaches have been shown to improve survival for patients with metastatic TNBC. With a deeper understanding of biological, molecular, immunological, microenvironmental, and clinical features novel agents have been developed to treat patients with TNBC; and promising agents are on the way (Fig. 5).

### Antibody drug conjugates (ADC)

It is becoming clear that ADCs are the wave of the future for how we can give chemotherapy (and other payloads) in a targeted fashion that minimizes the toxicities. These agents have changed the history of HER2+ breast cancer, and the ADC sacituzumab govitecan is effective in TNBC.

Sacituzumab govitecan is an ADC made up of an antibody against anti-human trophoblast cell-surface antigen 2 (TROP2) linked to the irinotecan derivative SN-38 as payload by a hydrolysable linker with a high drug-to-antibody ratio (DAR) of 7.5^67^. In the phase III ASCENT trial, this ADC improved progression-free survival (PFS) (5.6 vs 1.7 m) and OS (12.1 vs 6.7 m) compared to physician's choice chemotherapy in patients with metastatic TNBC who did not have brain metastases^68^. This benefit was, not surprisingly, correlated to TROP2 level expression on the tumor^69^.

Lots of other ADCs are in development (Table 1), and another TROP2-ADC, datopotamab deruxtecan (Dato-DXd, DS-1062a)^70^, demonstrated activity in a small cohort of pretreated TNBC, including those who had previously received sacituzumab govitecan, with an overall response rate (ORR) of 35%^71^. Similar ORR was seen with trastuzumab deruxtecan (T-DXd) in pre-treated patients with a new pathologic classification, “HER2-low”, meaning HER2-negative on clinical assays but with some low levels of expression (HER2 IHC score of 1+ or 2+/ISH negative) – 45% of which were TNBC^72^. The definitive phase III trial, DESTINY Breast 04 (DB04), compared T-DXd to treatment of physician’s choice (TPC), and recently demonstrated that T-DXd outperformed TPC in the ITT population, which included patients with HER2-low TNBC or hormone receptor-positive HER2-negative BC^73^. Most TNBC have high expression of LIV1, a protein key to transformation from an epithelial to a more motile mesenchymal cell. An ADC, ladiratuzumab vedotin (LV), is composed of anti-LIV1 antibody linked to an antimicrotubule agent, monomethyl auristatin E. In a phase 1 trial, LV resulted in an approximately 30% ORR in a heavily pretreated subgroup of TNBC patients^74^. Even higher ORR was reached by combining LV with pembrolizumab as first-line treatment in a very small cohort of TNBC patients^75^. Based on preclinical evidence of synergy between ADC and PARPi or ICI, other ongoing trials are focused on these combinations (Table 1).

**Table 1:** Ongoing trials of antibody drug conjugates in TNBC.

Drug	Target	Anti-body	Drug combinated	Study phase	Clinicaltrial.gov
Datopotamab deruxtecan (Dato-DXd)	TROP2	Datopotamab	Single agent	I	NCT03401385
			+ Durvalumab	I	NCT03742102 (ARM7)
			+AZD5305(oral PARPi)	I	NCT04644068 (Module5)
Sacituzumab govitecan (IMMU-132)	TROP2	Sacituzumab	Single agent	III	NCT04595565^a^
			Single agent	II	NCT04647916^b,c^
			+ Avelumab	II	NCT03971409
			+/− Pembrolizumab	II	NCT04468061
			+ Talazoparib	II	NCT04039230
			+ Atezolizumab	I/II	NCT03424005
Ladiratuzumab vedotin (SGN-LV1a)	LIV1	Ladiratuzumab	Single agent	I	NCT01969643(excluded part B and E)
			+ Pembrolizumab	I/II	NCT03310957
			+ Atezolizumab	I/II	NCT03424005
SKB264	TROP2		Single agent	I/II	NCT04152499
Patritumab Deruxtecan (U3-1402)	HER3	Patritumab	Single agent	II	NCT04965766
Trastuzumab Deruxtecan (T-DXd)	HER2	Trastuzumab	Single agent	Ib	NCT04556773^d^
			Single agent	III	NCT03734029^d^
			+ Pembrolizumab	I	NCT04042701^d^
			+ Chemo or immunoagents^e^	I	NCT04556773^d^
			+ Durvalumab	I	NCT03742102 (ARM6)
			+AZD5305(oral PARPi)	I	NCT04644068 (Module4)
Zilovertamab Vedotin (MK-2140) (VLS-101)	ROR1	Zilovertamab	Single agent	II	NCT04504916
Enfortumab Vedotin	nectin-4	Enfortumab	Single agent	II	NCT04225117 (Cohort 2)
SGN-CD228A	Melanotransferrin (CD228)	anti-CD228	Single agent	I	NCT04042480^c^
ASN004	5T4 oncofetal antigen (trophoblast glycoprotein)	Anti-5T4	Single agent	I	NCT04410224
CX-2009	CD166	Anti-CD166	Single agent	II	NCT04596150 (Arm B)
			+/−CX-072(pacmilimab)(PDL1i)	II	NCT04596150 (Arm C)
FDA018-ADC	TROP2	Anti-TROP2	Single agent	I	NCT05174637
OBT076 (MEN1309)	CD205	Anti-CD205	Single agent	I	NCT04064359^c^
MRG002	HER2	anti-HER2IgG1	Single agent	II	NCT04742153^d^
NBE-002	ROR1	Anti-ROR1	Single agent	I/II	NCT04441099
MORAb-202	Folate Receptor Alpha (FRα)	Anti-FRα	Single agent	I/II	NCT04300556
MGC018	B7-H3	Anti-B7-H3	+/− Anti-PD1 (MGA012)	II	NCT03729596
CAB-ROR2-ADC (BA3021)	CAB	Anti-CAR- ROR2	+/− Pembrolizumab	I/II	NCT03504488
PTK7-ADC (PF-06647020)	PTK7	Cofetuzumab	+ Gedatolisib	I	NCT03243331
Trastuzumab Duocarmazine (SYD985)	HER2	Trastuzumab	+ Paclitaxel	I	NCT04602117

^a^Post-neoadjuvant;

^b^brain metastases;

^c^in HER2-negative;

^d^HER2-low;

^e^Durvalumab, Paclitaxel, Capivasertib, Anastrozole, Fulvestrant, Capecitabine.

### Specific pathways targeting

DNA aberrations are common in TNBC, and many efforts have focused on targeting specific tumor pathways. Two promising phase II trials were designed to target the phosphoinositide-3-kinase (PI3K) pathway, altered in about 30% of TNBC with preclinical evidence of sensitivity to PI3K inhibitors. The two trials, LOTUS and PAKT, were phase II trials that combined paclitaxel with the AKT inhibitors ipatasertib and capivasertib. These trials demonstrated little benefit with addition of AKT-targeted agents in the overall population, but activity in PIK3CA/AKT/PTEN-altered TNBC^76,77^. However, in spite of this promise, the phase III IPAtunity130 trial did not confirm a benefit of ipatasertib added to paclitaxel as first-line treatment in TNBC with PI3KCA/AKT/PTEN alterations^78^; the analogous phase III trial of capivasertib has not yet reported.

Other studies were designed to target the EGFR signaling pathway that is almost universally activated in the BL-subtype, however an early trial, TBCRC 001, failed to demonstrate an effect of adding the anti-EGFR antibody cetuximab to carboplatin in TNBC patients^79^, and another randomized phase II trial demonstrated only a modest 10% improvement in ORR with the addition of cetuximab to cisplatin^80^. Serial tumor biopsies obtained in TBCRC 001 showed that the EGFR pathway was activated in most of these tumors, but the signaling was ineffective in some tumors, and there were alternative pathways to keep signaling intact despite EGFR inhibition in others^79^. RAS/RAF/MEK pathway activation is another common oncogenic driver. A MEK1/2-inhibitor (trametinib) window-of-opportunity trial in newly diagnosed TNBC demonstrated a strong treatment-adapted bypass response that involved multiple alternative tyrosine kinase signaling pathways^81^. These studies demonstrated that while there are strong preclinical data supporting targeting specific tumor pathways in TNBC, thus far in this highly mutable disease these efforts have not translated in clinical benefits.

### PARP inhibition (PARPi)

Germline (gBRCA1/2) or somatic BRCA1/2 mutation occurs in about 15–20% of TNBC^10^, with its hallmark ineffective homologous recombination DNA damage repair^82^ PARPi was first studied in previously treated metastatic patients with gBRCA1/2 mutation; just under half had TNBC^5^. Talazoparib improved median PFS by 3 months compared with chemotherapy of physician’s choice (TPC)^74^. The very similar OlympiAD trial compared olaparib to TPC in a similar population of largely pretreated gBRCA1/2-mutated metastatic patients with nearly identical results^4^. TPC in both trials did not include standard first-line drugs, particularly platinums, but regardless these lower-toxicity non-chemotherapy options are hugely valuable both in TNBC and non-TNBC gBRCA-associated breast cancer. Similar high responsiveness has been demonstrated with olaparib in the TBCRC 048 trial in germline PALB2 and somatic BRCA1/2^61^. Many efforts center on identifying the “BRCAness” phenotype among sporadic breast cancers with similarities to germline BRCA-associated and sensitivity to PARPi^83^. Previous PARPi trials in wildtype TNBC have been disappointing, but preclinical data suggest that combinations with ICI could overcome this limited activity. The phase II TOPACIO trial showed promising results (ORR 21%) with a combination of niraparib and pembrolizumab in a small cohort of TNBC of whom about half were germline carriers^84^; it remains unclear how much of the responsiveness was purely attributable to PARPi in gBRCA-associated tumors in that trial. There are several ongoing trials, such as EORTC-1984-BCG (NCT05209529) and DORA (NCT03167619), that will examine this potential synergy.

### Androgen receptor targeting

Various subtyping methods within TNBC consistently identify a subgroup with an expression of the androgen receptor pathway and more luminal features, often called the Luminal Androgen Receptor (AR) molecular subtype. There have been several trials in AR-positive BC (using multiple methods to define positivity), testing AR-inhibitors such as bicalutamide, abiraterone, enzalutamide, and seviteronel. All resulted in mostly clinical benefit (about 20%) as opposed to response^85–88^. These were all non-randomized trials, so it is hard to distinguish drug effects from natural history in this subgroup of TNBC, but this remains an area of active study and potential for a targeted approach.

### Immunotherapy in metastatic TNBC

As tumor aberrations are hard to target, a great deal of research focus has been on the interaction of cancer cells and the microenvironment^89^. ICI have demonstrated only modest activity as single agents in the metastatic setting, with up to 20% ORR in first-line and lower in later lines of therapy. Unlike early TNBC, and likely reflecting the more immune-suppressive metastatic microenvironment, PDL1 status is predictive of benefit from ICIs. Real benefit of these drugs is limited to the first-line setting and in combination with chemotherapy. The practice changing trial was IMpassion130, which randomized 902 untreated advanced TNBC patients to receive nab-paclitaxel plus atezolizumab (antiPDL1) or placebo^6^, which revealed PFS and OS benefits of 2.5 and 7.5 months, respectively, limited to the PDL1+ subgroup defined as ≥1% PD-L1 expression by Ventana SP142 assay^90^. On the basis of this trial, atezolizumab received accelerated approval from the FDA in 2019, however, this indication was withdrawn in 2021 by the sponsor after the negative phase III IMpassion131, which was similar to IMpassion 130 but with a paclitaxel backbone^91^. Adding pembrolizumab to several chemotherapy regimens (nab-paclitaxel, paclitaxel, or gemcitabine/carboplatin) in KEYNOTE 355 improved PFS and OS in chemo-naive-PDL1+ TNBC patients, defined as CPS ≥ 10% PDL1-expession by the Dako 22C3 assay, including patients who relapsed early after adjuvant chemotherapy^92^. In no trial has an impact of immunotherapy in PDL1-negative metastatic patients been observed, and the best chemotherapy combination with ICI remains an open question. Other immunotherapy approaches such as vaccines, cell therapies and new combinations are being developed to hopefully enlarge the TNBC population who could benefit from immune-based therapy.

## TNBC in the community

### Racial disparities in outcomes and TNBC

As noted, TNBC is more frequent among Black and young women with BC. Using molecular profiling applied to the CBCS dataset, even more striking differences by race are seen; for example, a Black woman under 50 years old at diagnosis has a 37% chance of having Basal-like breast cancer and only 25% chance of having the good-prognosis Luminal A, whereas a White woman over 50 years old has only a 15% chance of having Basal-like and 52% chance of having a Luminal A tumor^46^.

Given that major advances in treatment have largely been seen in the hormone receptor- and HER2-positive clinical subtypes, it is not surprising that TNBC has the poorest prognosis of all BC, contributing to worse outcomes in Black women simply due to the frequency of this subtype^39^. It should be noted that TNBC carries a poorer prognosis in both Black and White women; within-subtype racial disparities are actually greatest among hormone receptor-positive, HER2-negative BC^93^.

### Burden of TNBC

Patients with cancer have to face many concerns after their diagnosis, including costs, the important decision of where and how to be treated, and what can be expected in their future. Beyond the psychological aspects of facing a life-threatening disease and treatment toxicities, another burden of this disease is represented by financial toxicity that particularly affects patients of color across all tumor types^94^, and is associated with lower quality of life, distress, treatment delays or discontinuation, and higher mortality^95^ . Within two years of diagnosis, the negative impact of financial costs is quite substantial as self-reported by all patients with BC enrolled in phase 3 of the CBCS, but even more striking among Black women (58 vs 39% of White women), including income loss, healthcare-related financial and transportation barriers, job loss, and loss of health insurance. Those who were young and those who received chemotherapy, both factors likely enriching further for TNBC, were 10% more likely to experience adverse financial impact^96^. Specifically looking at cancer-related job or income loss, which was reported by 38% of over 2400 CBCS3 participants, Black women and women living in rural neighborhoods all saw greater income reduction from their cancer diagnosis than urban White women^97^. These financial adverse events have to be kept in mind due to their impact on treatment choice, compliance, well-being, and on cancer outcomes.

The umbrella term “TNBC” represents a spectrum of tumors that are disparate both from a tumor and from a microenvironmental standpoint. With advances in chemotherapy, and more recently immune checkpoint inhibition, cure rates have improved in early TNBC and are likely to accelerate. Outcomes in metastatic BC are already being altered by new drugs (ADC, PARPi, ICI) but big transformations are still needed. New ideas are emerging that may allow tumor targeting (e.g., epigenetic modulators preventing reprogramming, AR targeting), prevent immune evasion (e.g., DDR1 antibodies, combinatorial immunotherapy) and maybe even prevent TNBC. In addition to considering the need for better and more precise treatment, we must also consider all disease-related burdens, mindful that while TNBC can occur in anyone, it does particularly affect young and Black women and contributes to all kinds of poorer outcomes. The focus must remain on improving treatment, better managing toxicity, and social and financial support for patients with TNBC.

## Data Availability

Source data for all tables are provided in the paper. No new datasets have been generated or analyzed for this article.
